# Titanium membrane layered between fluvastatin-loaded poly (lactic-co-glycolic) acid for guided bone regeneration

**DOI:** 10.1093/rb/rbac061

**Published:** 2022-09-06

**Authors:** Akihiro Furuhashi, Yunia Dwi Rakhmatia, Yasunori Ayukawa, Kiyoshi Koyano

**Affiliations:** Section of Implant and Rehabilitative Dentistry, Division of Oral Rehabilitation, Faculty of Dental Science, Kyushu University, Higashi, Fukuoka-ku 812-8582, Japan; Section of Implant and Rehabilitative Dentistry, Division of Oral Rehabilitation, Faculty of Dental Science, Kyushu University, Higashi, Fukuoka-ku 812-8582, Japan; Department of Prosthodontic, Faculty of Dentistry, Padjadjaran University, Kota Bandung, Jawa Barat 40132, Indonesia; Section of Implant and Rehabilitative Dentistry, Division of Oral Rehabilitation, Faculty of Dental Science, Kyushu University, Higashi, Fukuoka-ku 812-8582, Japan; Division of Advanced Dental Devices and Therapeutics, Faculty of Dental Science, Kyushu University, Higashi, Fukuoka-ku 812-8582, Japan

**Keywords:** titanium mesh, PLGA, statin, guided bone regeneration (GBR)

## Abstract

The aim of this study was to investigate titanium membranes (TMs) layered between poly (lactic-co-glycolic acid) (PLGA) containing fluvastatin (FS) for use in guided bone regeneration. Membranes consisting of PLGA, FS-containing PLGA (PLGA–FS), TM layered between PLGA (TM–PLGA) and TM layered between FS-containing PLGA (TM–PLGA–FS) were prepared, and their mechanical and chemical properties were evaluated. The TM groups showed statistically significant differences, in terms of tensile strength and elastic modulus, when compared to the PLGA groups. The release of FS was demonstrated to be higher in the TM–PLGA–FS group than the PLGA–FS group after Day 14. The effect of membrane implantation on the calvaria of Wistar rats was measured using micro-computed tomography (micro-CT) and morphometrical analyses, as well as histological observations. At 4 weeks, the TM–PLGA–FS and TM–PLGA groups were found to have lower bone mineral density but higher bone formation, when compared to the control and PLGA groups. At 8 weeks, the use of TM–PLGA–FS membranes significantly enhanced bone formation in the calvaria model, compared to the other groups. These results suggest that a TM layered between PLGA containing FS potentially enhances bone formation, thus showing good potential as a GBR membrane.

## Graphical Abstract

**Figure:**
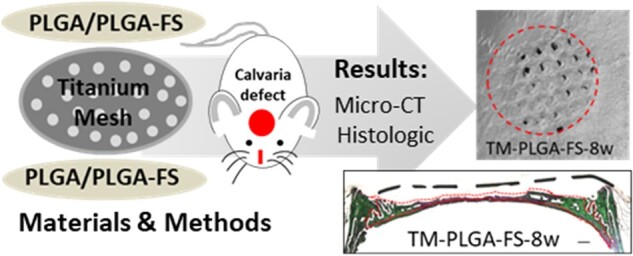

## Introduction

Various membranes have been developed for bone augmentation, which can be divided into degradable and non-degradable membranes. These membranes should fulfill the requirements for guided bone regeneration (GBR), including biocompatibility, space preservation, cell occlusivity and clinical manageability. Titanium membranes (TMs) are non-degradable membranes that have excellent mechanical properties to augment new bone. They provide spatial maintenance, prevent membrane collapse into the specific space due to mucosal compression, prevent graft displacement, and permit handling and adaptation to a bony defect [1, 2]. Despite their success related to their stiffness (which maintains the structure and prevents the membrane from collapsing), TMs have drawbacks, such as mechanical irritation of the mucosal flaps, membrane exposure and soft tissue ingrowth [3–5]. Other features of TMs include their macro- and multi-porosity, which are related to the sharp edges caused by cutting, bending or trimming, possibly leading to soft tissue damage following membrane exposure, thus raising complications of membrane treatment failure [6]. In addition, various factors have been reported, in terms of determining the amount of bone formation underneath the membrane, including physical and biological properties, the chemical composition of the material, and the location at which the membrane is placed within the tissue [7]. The infiltration of connective tissue cells or fibroblasts to the defect area should be hindered, as they may prevent the function of the membrane; however, our prior *in vitro* work has demonstrated that membranes with macro- and multi-pores allow for the penetration of fibroblasts through their pores [8]. The macroporosity of TMs can lead to soft tissue penetrating the defect through the pores and, thus, the membranes are difficult to remove in the follow-up surgery. In contrast, another study has suggested that selectively permeable membrane barriers are necessary for nutrition and blood diffusion to the defect area and the surrounding tissue, especially during the early stages of healing [9]. Thus, the development of less porous and microporous TMs could alleviate the current difficulties associated with TMs.

Poly (lactic-co-glycolic acid) (PLGA) is a membrane that can be resorbed in the body, thus eliminating the need for second-stage removal surgery. PLGA possesses the characteristics of flexibility and biocompatibility, and has been used for the controlled administration of drugs, peptides and proteins [10]. However, PLGA membranes are not stiff enough to resist soft-tissue pressure during healing and have unpredictable degradation patterns, which can significantly alter the results of GBR. Membrane collapse is one of the observed complications during GBR, which reduces the volume of the bone to be augmented and decreases restoration of the original bone shape [11, 12]. Appropriate mechanical, physical and bioactive properties are required of the membrane [13]; however, the biodegradability rate is also an important factor as, at most, 6 months of bone healing are required for successful regeneration [14, 15].

Statins are hydroxymethylglutaryl–coenzyme A reductase inhibitors, which have been widely used as hypercholesterolemia drugs. Statins have been reported to accelerate hard and soft tissue healing in the oral cavity [16], enhance vascular endothelial growth factor production [17], improve wound healing and exhibit antimicrobial effects [18]. Thus, statins have the potential to enhance bone formation, in terms of both volume and mineralization. Previous studies have reported research on the use of statins as bio-stimulus agents, through systemic administration or topical application, in order to observe bone formation and soft tissue healing around implants [18–22]. Systemic administration is a method of administering statins into the circulatory system, such that the entire body is affected. Statin administration can take place via an enteral route (i.e. where the drug is absorbed through the gastrointestinal tract) or parenteral administration (i.e. through injection, infusion, or implantation) [19]. In contrast, topical administration involves drugs that are applied to the designated area with local effects [20–22]. However, an appropriate drug is essential in the drug delivery system (DDS) to obtain a clinically successful outcome. In this study, statins were not administered systemically or topically but, instead, loaded in PLGA membranes for GBR treatment.

Our previous study has shown that fluvastatin (FS)-loaded degradable PLGA has the potential effect to enhance soft and hard tissue healing in the tibia of rats [23]. However, the study revealed only minimal bone formation in a critical size defect site of the rats. This may be due to the mechanical stability of PLGA membranes, which was not stiff enough to support the space to be maintained.

This study was conducted to improve the predictability and to resolve the problems related to FS-loaded PLGA membranes and TMs. Despite the disadvantage of non-resorbability, meaning that the membrane must be removed after bone regeneration, we fabricated a FS-loaded PLGA reinforced with a TM. The PLGA membrane was expected to cover the sharp and rough edges of the TM after bending, cutting and/or trimming. FS-loaded PLGA was also used, in order to avoid soft tissue ingrowth through the large pore size of the TM and to alleviate the difficulties associated with membrane removal. In addition, by using PLGA membranes loaded with statins, we could take advantage of the positive effects of the statins, as mentioned above.

## Materials and methods

### Fabrication of membranes

The commercially available titanium mesh used in this study was Jeil Titanium Mesh (Proseed Corporation, Tokyo, Japan). PLGA was purchased from Wako Pure Chemical Industries (Osaka, Japan). Dichloromethane and polyvinyl alcohol were purchased from Nacalai Tesque (Kyoto, Japan). FS was obtained from Toronto Research Chemicals (North York, Canada).

PLGA membrane loaded with or without FS was fabricated as described in a previous report [23]. In short, to prepare a single-phase solution, 2.4 g of PLGA was dissolved in 3 ml dichloromethane. The solution was then mixed without FS (PLGA group) or mixed with 24 mg of FS (PLGA–FS group). Then, the PLGA mixture was emulsified in polyvinyl alcohol with stirring to evaporate at 60°C for 3 days. The mixture was dropped onto silicone rubber, in order to ensure easy membrane removal, pressed using another silicone rubber on the mixture, and dried in a vacuum oven at 37°C for 1 day. To form the PLGA-layered TM, a TM was put between the two layers of PLGA. Then, the membrane was pressed and dried in an oven at 37°C for 1 day. The titanium-layered PLGA was divided into two groups: the TM–PLGA group and the TM–PLGA–FS group. The thicknesses of all membranes were measured at four points per piece of membrane using a digital micrometer (*N* = 5).

### Morphological surface analysis

Morphological surfaces of all sample membranes were evaluated using a scanning electron microscope (SEM; S-4800; Hitachi, Tokyo, Japan). The membrane samples were coated with gold before they were connected to a short column and placed in the vacuum chamber of the SEM. The chamber was set at an accelerating voltage of 10 kV and the surface microstructures were photographed at a magnification of 1000×.

### Mechanical test

The mechanical properties of the membranes were evaluated to measure the stress–strain value, the tensile strength and the elastic modulus of experimental membranes (*N* = 5) using a universal testing machine (AG-IS, Shimadzu, Kyoto, Japan). Membrane specimens with size of 20 × 25 mm were prepared and attached to holders equipped with a 50 kgf load cell and a cross-head speed of 50 mm/min.

### pH measurements

Membranes with a diameter of 8 mm were suspended in 50 ml of saline solution in conical tubes under a pH of 7.4 at 37°C. Slight changes in the pH of the solution containing the membranes were measured every 7 days using a pH meter. Each type of experimental membrane was replicated three times.

### Fluvastatin release test

The cumulative release rate of the FS from the PLGA–FS and TM–PLGA–FS membranes was determined using a photometric assay. Membranes with a diameter of 8 mm were suspended in 50 ml of PBS at 37°C. PBS supernatant (70 μl) was prepared and analyzed daily by spectrophotometry at 238 nm for 30 days. The mean values of three replicates of PLGA–FS and TM–PLGA–FS membranes were used to compare their cumulative release rate. Standard concentrations of FS were prepared, in order to determine the standard curve, and the absorbance values were measured. According to the standard curve, the FS release was measured using the following equation: *y* = 1.7115*x* + 0.1694 (*R*^2^ = 0.9695), where *x* is the concentration of FS (mg/ml) and *y* is the absolute absorbance value of the FS at 238 nm [24].

### Evaluation of membranes in rats

A total of 50 eight-week-old male Wistar rats were purchased from Kyudo (Tosu, Japan) and used in the present study. The rats were treated in accordance with guidelines for animal care from The Institutional Animal Care and Use Committee at Kyushu University (Fukuoka, Japan) (approval number: A29-195-0). All animals were placed under identical conditions and fed a commercially available standard rodent food (CE-2, CLEA Japan, Tokyo, Japan), with water available ad libitum. The animals were divided into five groups—PLGA, PLGA–FS, TM–PLGA, and TM–PLGA–FS, and a control (C)—with two healing times (4 and 8 weeks). Each group consisted of five animals.

### Surgical procedures

**Figure 1.**
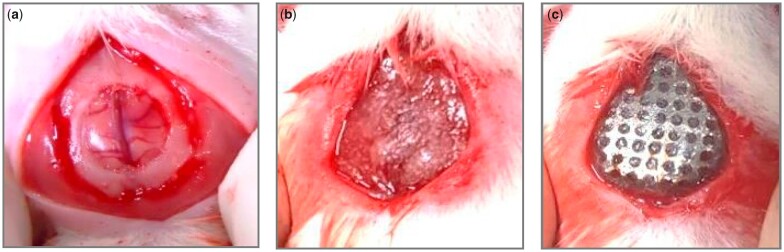
Surgical procedure: (**a**) a circular 8 mm defect was created on rat calvaria; and (**b**) PLGA or (**c**) TM–PLGA–FS was placed on the defect site.

The head of each animal was shaved and disinfected with iodine. Then, an incision was made with a scalpel to reveal the parietal bones. A circular defect 8 mm in diameter was created on the rat’s calvaria. The defect site was either covered with a membrane or left untreated, the latter of which was used as a control (Fig. 1). The surgical site was then sutured and closed.

### Radiographic images and micro-computed tomography analysis

At 4 and 8 weeks after implantation, all animals were euthanized and an excision biopsy of the calvaria area, including the membrane and adjacent hard tissue, was dissected. The unprocessed bone biopsies were immersed in paraformaldehyde solution for 24 h and scanned using an *in vivo* micro-computed tomography (micro-CT) scanner (SkyScan 1076; SkyScan, Aartselaar, Belgium). Specimens with slice thickness of 18 μm were scanned. Reconstruction was accomplished using the Skyscan NRecon software. Radiographic images of the middle section and 3D reconstruction images were observed to assess bone regeneration in all samples. The bone mineral density (BMD; g/cm^3^), the three-dimensional (3D) bone volume (%) and the two-dimensional (2D) bone area (mm^2^) in the middle-most section were measured using micro-CT analysis software (CTAn; SkyScan).

### Morphometric and histological evaluation

The excised samples were dehydrated and embedded into methacrylate resin (Nacalai Tesque). Undecalcified sagittal sections were cut, polished and stained using Masson’s trichrome method. Bone formation in the defect site and the adjacent bone were observed histologically and measured morphometrically using a light microscope (BioRevo BZ-9000; Keyence, Osaka, Japan). The centers of all samples from the histological sections were selected to represent the respective group for evaluation. To measure the areas of new bone formation, the sections were observed at 10× magnification. Demarcation lines were made to indicate the edge between the original bone walls and the new bone areas.

### Statistical analysis

The cumulative release rate between PLGA–FS and TM–PLGA–FS was analyzed using paired t-test analysis. A one-way analysis of variance was performed to analyze the tensile strength, elastic modulus, pH, BMD, 3D bone volume, 2D bone area and morphometric bone area, along with Tukey’s post-hoc test (SPSS 12.0 J, SPSS Japan, Tokyo, Japan). Differences among the groups were considered significant at *P* < 0.05.

## Results

### Membrane characteristics

The PLGA and PLGA–FS membranes, and PLGA or PLGA-FS layer covering TM were flexible and translucent. Yellow granules of FS powder were observed in the PLGA–FS and TM–PLGA–FS groups. The mean thicknesses of the PLGA, PLGA–FS, TM–PLGA and TM–PLGA–FS membranes were 0.78, 0.79, 0.98 and 1.00 mm, respectively.

**Figure 2.**
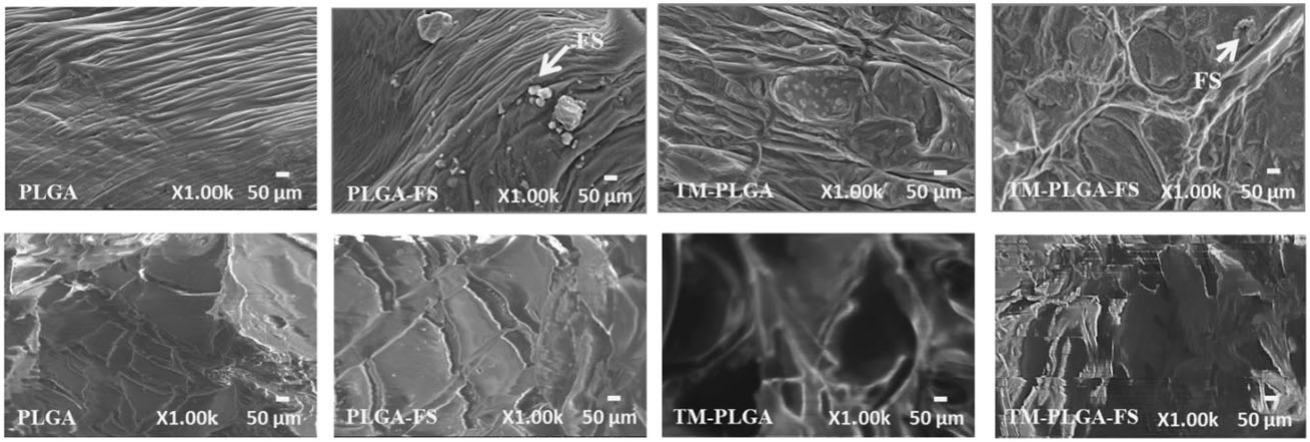
Membrane surface analysis by SEM (top) and cross-section topography (bottom). All membranes appeared dense and flat with some folds. FS granule was observed in PLGA–FS and TM–PLGA–FS groups. Circle traces of TM macropores were observed in the TM–PLGA and TM–PLGA–FS groups. Magnification: 1000×, scale bar: 50 μm.

The surface topographies of all membranes were observed under SEM at 1000× magnification (Fig. 2). Macroscopy and morphology were analyzed, and no obvious differences were observed among the membranes. The SEM images indicate that the surfaces of all the membranes were dense and flat with some folds. However, the TM–PLGA and TM–PLGA–FS membranes were observed to be rougher than the PLGA membranes, and circle traces of TM pores could be seen on their surfaces. FS particles were observed to be embedded in the folds of the PLGA–FS and TM–PLGA–FS membranes.

### Mechanical test

**Figure 3.**
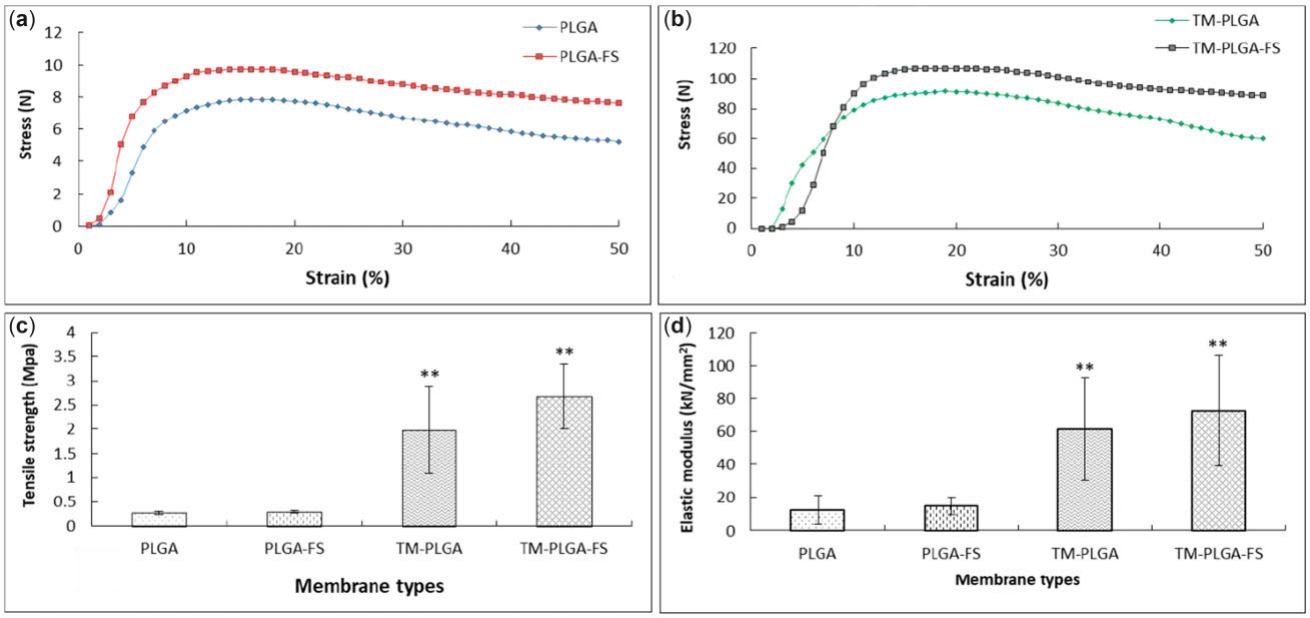
Mechanical evaluation of all membranes: (**a**) stress–strain curves of PLGA and PLGA–FS membranes; (**b**) stress–strain curves of TM–PLGA and TM–PLGA–FS membranes; (**c**) tensile strength analysis; and (**d**) elastic modulus (*P* < 0.05; **compared to PLGA and PLGA–FS).

The results of the mechanical tests for all membranes are shown in Fig. 3. In Fig. 3a, the stress–strain curve of PLGA can be seen to be lower than that of the PLGA–FS membrane. In Fig. 3b, the stress–strain curve of the TM–PLGA membrane is shown to be lower than the TM–PLGA–FS membrane at a strain of 8%. The TM groups showed higher stress–strain curve values, higher tensile strengths and elastic modulus, with statistically significant differences when compared to the PLGA groups. However, no significant differences were found between the tensile strengths of the PLGA and PLGA–FS groups or the TM–PLGA and TM–PLGA–FS groups, respectively.

### pH measurement

**Figure 4.**
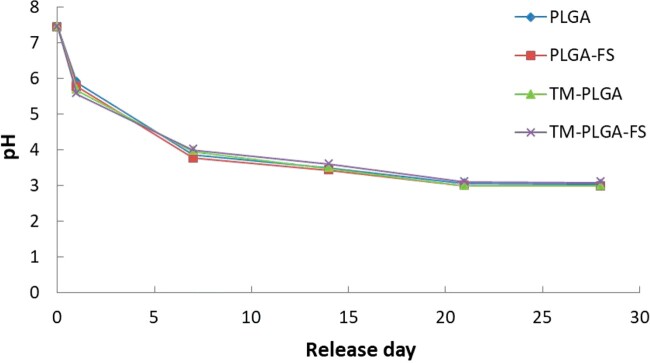
pH measurements of all membranes at 0, 1, 7, 14, 21 and 28 days.

All membranes showed similar changes in pH after 28 days of measurement (Fig. 4). The pH gradually decreased at 7, 14 and 21 days, and was relatively constant after 21–28 days. On Day 1, the pH of the saline solution had decreased to a pH of about 5.5, continued to decrease until 21 days to a pH of about 3, and then remained relatively constant after 21 days at a pH of 3.

### Spectrophotometric analysis

**Figure 5.**
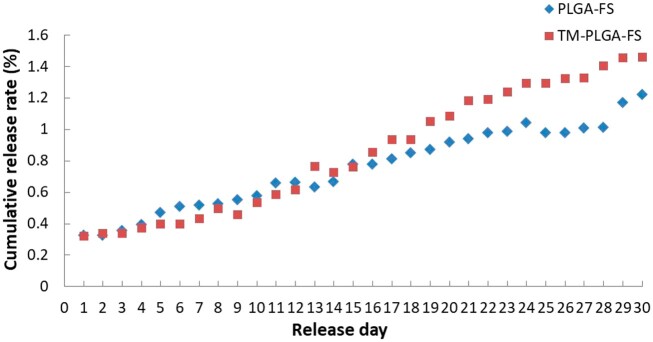
Cumulative release rates of FS from PLGA–FS and TM–PLGA–FS membranes in 30 days.

FS encapsulated in the PLGA–FS and TM–PLGA–FS membranes was gradually released into 50 ml PBS solution and analyzed daily for 30 days, as shown in Fig. 5. The cumulative release rate of FS was observed to be 0.3% on Day 1. After Day 14, the release of FS was demonstrated to be higher in the TM–PLGA–FS group compared to the PLGA–FS group. The total FS released from the PLGA–FS and TM–PLGA–FS groups was 4.78 and 5.86 μg/50 ml, respectively.

### Radiographic and 3D reconstruction images

**Figure 6.**
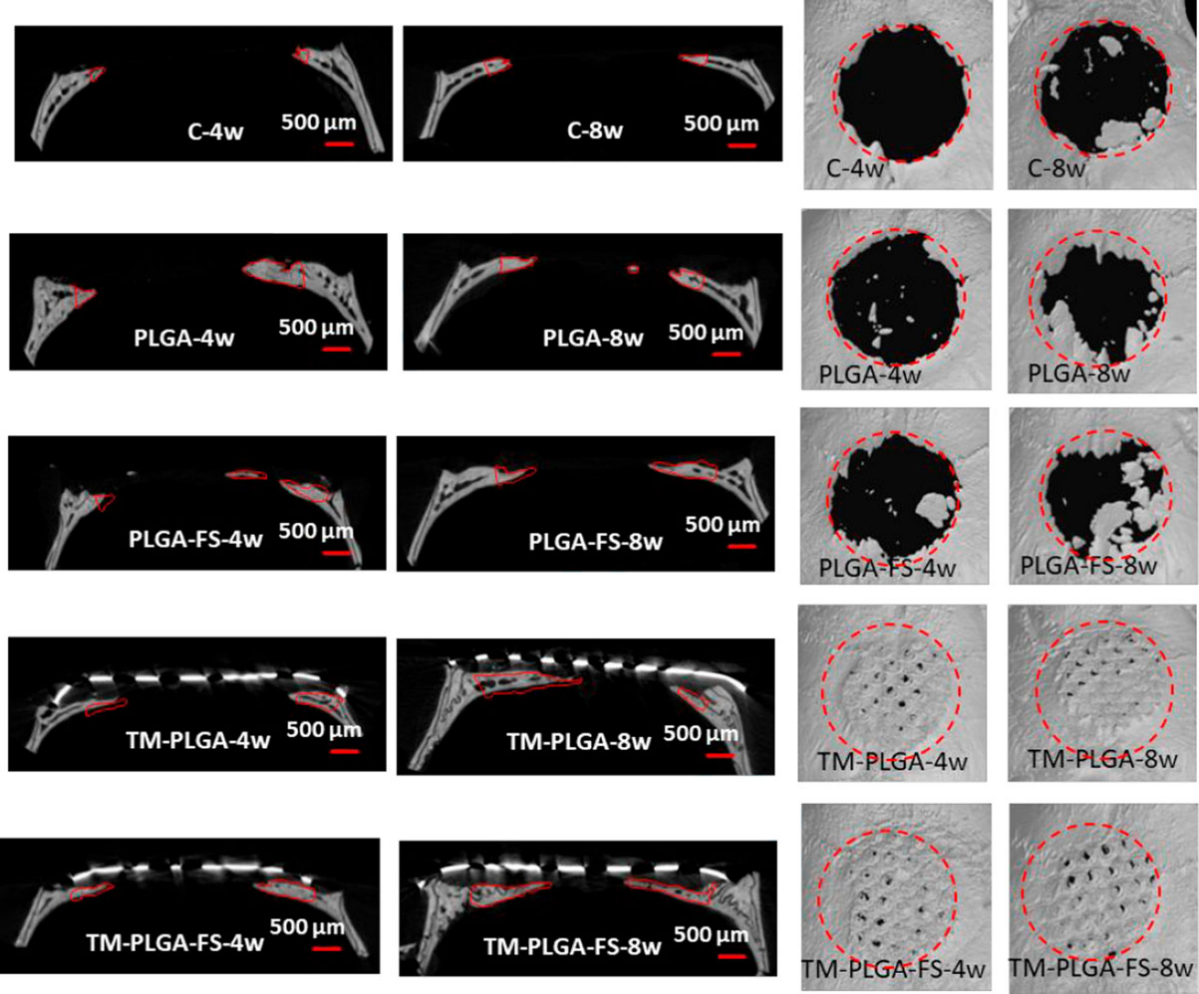
Radiographic (middle section) and 3D reconstruction images of all membrane groups at 4 and 8 weeks (*N* = 5). Minimal bone formation was observed in C and PLGA groups, while more bone formation was observed in the PLGA–FS, TM–PLGA and TM–PLGA–FS groups (red line). The amount of newly formed bone was found to be larger at 8 weeks, compared to 4 weeks.

Radiographic and 3D reconstruction images of representative samples are shown in Fig. 6. As can be seen from the figures, all groups showed more bone formation after 8 weeks, compared to 4 weeks. The TM–PLGA–FS groups presented the highest bone formation, compared to the other groups. After 8 weeks, the defects in this group had almost closed, compared to the other groups.

### Micro-CT and morphometric analyses

**Figure 7.**
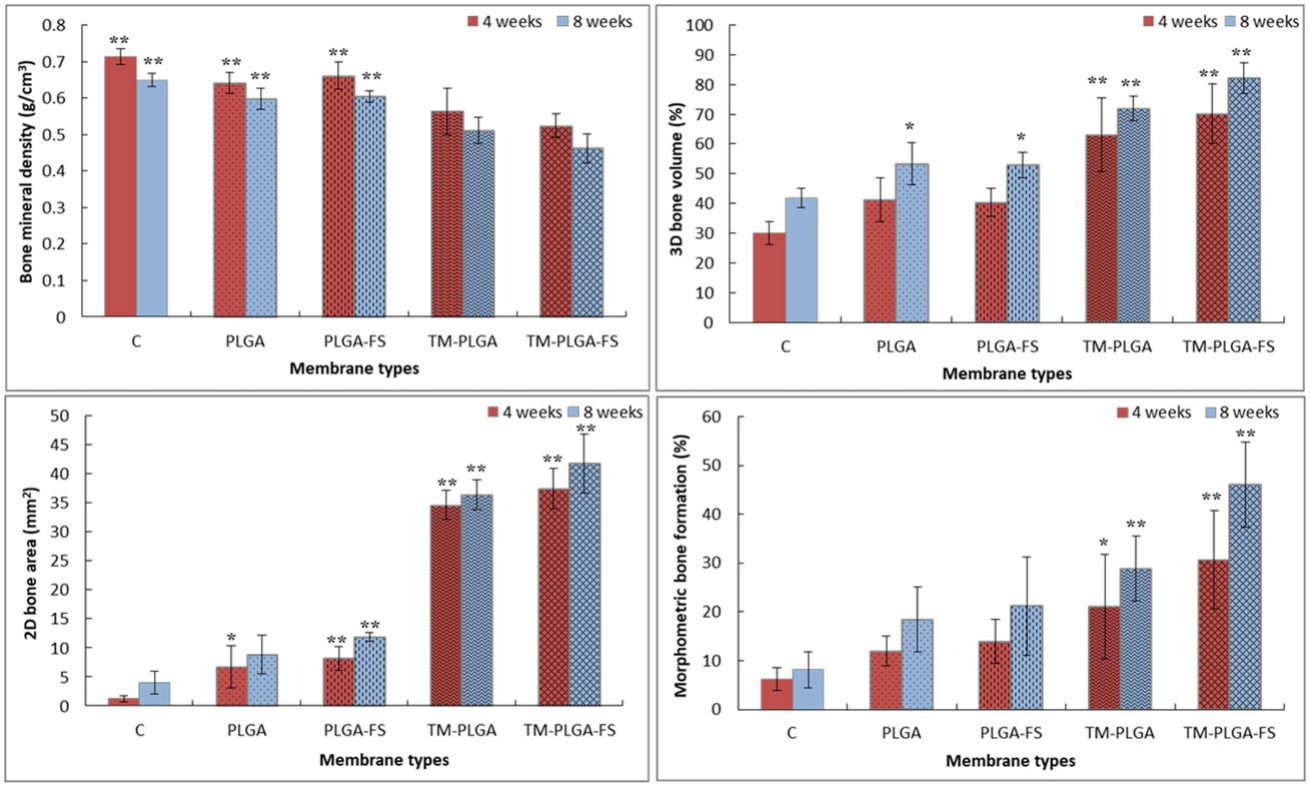
Micro-CT analyses of BMD, 3D bone volume, 2D bone area and morphometric bone area of all membrane groups at 4 and 8 weeks (*N* = 5). New bone formation was higher in TM–PLGA–FS compared to other groups. BMD in both TM groups were observed to be lower, compared to other groups.

The BMD, 3D bone volumes and 2D bone areas were observed through micro-CT analyses and morphometric bone formation was measured using histological sections, as shown in Fig. 7. From the analyses, at 4 weeks, the TM–PLGA–FS and TM–PLGA groups were seen to have lower BMD but higher bone formation, as shown by the 3D bone volume and the 2D bone area, compared to the C, PLGA and PLGA–FS groups (*P* < 0.01). No significant differences were found between the TM–PLGA and TM–PLGA–FS groups in bone volume, BMD, 2D bone area and morphometric bone formation at 4 weeks. However, the TM–PLGA–FS groups was observed to have significantly higher bone volume and morphometric bone formation, compared to the TM–PLGA group (*P* < 0.05), at 8 weeks.

### Histological analysis

**Figure 8.**
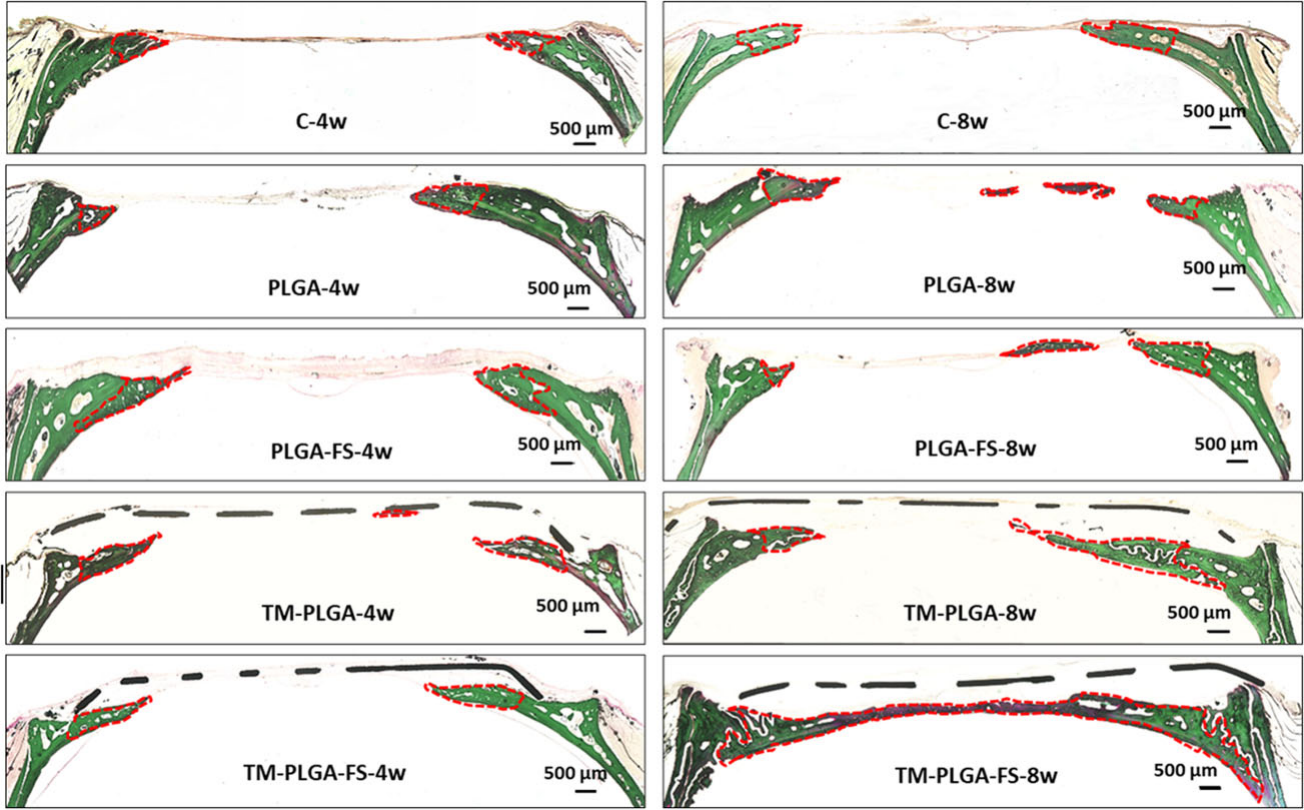
Histological analyses of all membrane groups at 4 and 8 weeks. All groups show various amounts of new bone formation (red line). New bone was observed to be larger at 8 weeks than at 4 weeks. TM–PLGA and TM–PLGA–FS presented abundant new bone formation, compared to PLGA, PLGA–FS and C groups at 8 weeks. Magnification: 4×, scale bar: 500 μm.

At week 4, minimal bone formation was observed in the C group, while some bone formation was observed in the experimental groups (Fig. 8). The bones were formed adjacent to the original bones in the defect areas, but remaining PLGA or PLGA–FS membranes were not observed. In these groups, the defect areas were covered with thick connective tissue.

At week 8, larger bone formations were observed adjacent to the original bones and more in the center of the defect than the samples in the groups at 4 weeks. Abundant bone formation was present in the TM–PLGA and TM–PLGA–FS groups. In some samples, newly formed bone had almost covered the defect areas in the TM–PLGA–FS group.

## Discussion

The physical and biological properties of biomaterials influence their function, and the selection of a specific material is based on the treatment requirements, as well as the inherent advantages and disadvantages of each material [25]. In terms of degradable membranes, the biodegradability rate is an important parameter [26], along with the appropriate mechanical, physical and bioactive properties of the membrane [27]. In our previous study, PLGA membranes loaded with FS were implanted on rat calvaria to increase their ability to regenerate new bone formation, especially in the initial period of healing. However, we found that only minimal bone had formed underneath this membrane, and the PLGA membrane had almost degraded after 8 weeks of healing time [23]. Despite the necessity of a second-stage surgical procedure for membrane removal, TMs may overcome the limitations of degradable membranes. They are more predictable in their performance, maintaining a larger defect space, with long-term survival and high success rates [28, 29].

In this study, a commercially available TM was used to reinforce PLGA and FS-loaded PLGA membranes, which successfully induced and increased bone formation. Treatments for bonding between polymers and TM using etchants, chemical methods, or mechanical surface roughness techniques may affect the surface brittleness and create micro-hardness regions susceptible to bacterial attachment [30]. A study has reported that the solution diffusion model was suitable for loading drug particles into polymeric membrane surfaces [31]. In addition, titanium particles were found to inhibit osteoblast activity, suppress their differentiation and induce apoptosis [32–34]. Another study has reported that direct exposure to titanium particles was found to enhance osteoclast activity at a low concentration [35]. However, PLGA layers covering TM may prevent the exposure of titanium particles, inhibiting the results of bone formation. Similarly, a previous study using PLGA film with 250 nm thickness found that it could improve osteoblast activity [36].

In the PLGA groups, the membranes could maintain space initially, but generally lost strength and collapsed into the defect, leading to failed regeneration. A similar previous study has reported that degradable membranes may tend to collapse when treating periodontal defects [37]. A GBR membrane should fulfill a certain level of mechanical stability, in order to maintain the space and prevent soft tissue ingrowth at the defect site. The mechanical properties of the drug-incorporated PLGA membrane may be used to tune the materials and their interaction, drug release behavior, and polymer degradation. Several factors appear to influence the mechanical properties of drug-loaded polymers, including molecular weight, the polymer composition, loading processing, temperature and concentration of drugs. The design and function of drug-loaded polymers may affect their strength and stiffness, due to the porous structure [38]. Small-molecule drugs in electrospun polymer fibers resulted in lower crystallinity of polyester and polyether materials [39]. Polymer fibers also have the potential to adjust drug miscibility, and the drug-loaded polymer interaction could lead to different release profiles [40]. However, the effects of FS-loaded PLGA were observed through the higher stress–strain curves. It can be seen that the PLGA–FS had little difference in the deformation mechanism, and the stress–strain measurements of PLGA–FS tended to rise, due to changes in sample porosity. Meanwhile, the PLGA membrane showed a decline, as a consequence of the degradation process. In contrast, a study has reported that the thickness of samples may lead to incomplete removal of the residual solvent by evaporation during solvent casting [41].

The tensile strengths in this study for the PLGA, PLGA–FS, TM–PLGA and TM–PLGA–FS samples were 0.27, 0.29, 1.98 and 2.67 MPa, respectively. The tensile strength of the TM without PLGA or the PLGA–FS layer had the highest tensile strength, among other membranes. Therefore, the PLGA and PLGA–FS layers covering the TM mesh seemed to influence the tensile strength of the TM. The PLGA and PLGA–FS membranes used in this study had lower tensile strengths, compared to those in a previous study using a PCL/PLGA hybrid membrane, which presented 2 MPa in the dried state and 1.5 MPa in the wetted state; however, a tensile strength of more than 15 MPa in the dried membrane was also reported [42]. In contrast, a calcium alginate membrane with tensile strength of 0.017 MPa has been reported to produce perfect recovery in a rat tibia model at 8 weeks [43]. A previous study has reported that the tensile strength of a polycaprolactone membrane incorporating drugs with nanohydroxyapatite was lower than that of the polycaprolactone membrane only [44]. In contrast, our previous study has reported that the tensile strength of membrane containing drugs (PLGA–FS) were higher than the membrane without drugs [23]. However, the tensile strength was found to not significantly differ between the membranes with and without drugs. Various tensile strengths of membranes used for GBR have been reported, but a range of ideal tensile strengths has not yet been defined. However, high tensile strength is an important characteristic ensuring the mechanical stability of membranes in GBR treatment. The elastic moduli of PLGA, PLGA–FS, TM–PLGA and TM–PLGA–FS samples were 12.50, 14.85, 61.68 and 72.68 kN/mm^2^, respectively, where a higher elastic modulus indicates a hardening effect. The elastic modulus values of FS-loaded membranes were also observed to be higher, when compared to membranes without FS. Thus, the addition of FS affected the mechanical properties of the membranes, whose tensile strength and elastic modulus were higher than that of the PLGA membrane groups. An increase in elastic modulus may influence the interaction of drug particles with the polymer chains [45].

Rat calvaria were used to evaluate the membranes in GBR because they have a poor blood supply and their structure has low mechanical stimulation that precludes any spontaneous healing [46]. A study has found that the smallest defect size of 5 mm in rat calvaria does not heal completely during the lifetime of the animal [47], although another study has estimated this to be larger, at 8 mm in diameter [48]. In the present study, in the PLGA membrane groups, membranes were still present at 4 weeks but had almost completely degraded after 8 weeks of healing time. The biodegradable membranes must provide mechanical support and a suitable degradation process, such that the bone tissues and soft tissues have a period of healing and growth in the defect area. If a membrane has a degradation process faster than the bone formation process, it may prevent bone formation and allow soft tissue ingrowth in the defect area. In contrast, the TM groups were still intact with the adjacent bone, thus covering the defect, although the layers of PLGA or PLGA–FS had almost completely degraded by 8 weeks. However, the function of the TM—that is, to keep the defect away from connective tissue ingrowth—was still maintained. The layers of PLGA or PLGA–FS prevented the ingrowth of soft connective tissue to penetrate through the macropores of the TMs and, thus, membrane removal may be easier in clinical applications.

Our *in vitro* pH measurement study indicated the acidic environment of all experimental membranes. After 28 days, the pH of PLGA, PLGA–FS, TM–PLGA and TM–PLGA–FS samples was 3.03, 2.99, 2.98 and 3.08, respectively. However, no significant difference was found in pH between the membranes. A study has reported that the microenvironment pH sensed by the dye over a broadly acidic was in the range of 2.8–5.8 [49]. In PLGA delivery systems, the pH has been found to be more acidic due to the polymeric quantity, composition, molecular weight and variable lactic/glycolic acid ratio [49, 50]. The hydrolysis rate of the polymer and the accumulation of acidic polymer degradation products affected the lower pH in the microenvironment during 4 weeks of incubation [49]. Previous studies have implicated the instability of encapsulated acid-labile proteins in influencing the acidic microenvironment [51–53]. In addition, an acidic microenvironment containing PLGA degradation products significantly inhibited the viability and biological behaviors of cells [54]. The acidosis environment increases the activity of the osteoclasts and decreases activity of the osteoblasts [25]. Specific conditions, such as an appropriate amount of polymer materials and the dosage of PLGA, or a complementary combination of other biomaterials, may be key factors affecting regeneration effects. A study has reported that the degradation products of combined PLGA and chitosan in the microenvironment reduced the inflammatory response and enhanced cellular regeneration [54]. Further studies observing the possible adverse effects of acid degradation products of PLGA and their impact on regeneration are needed for comprehensive observations and evaluations.

Successful treatment of DDS depends on the correct amount and the appropriate delivery of drug carriers. Drug carriers that are incorporated with a substance can be used to prolong the delivery and effectiveness of drugs. Generally, the interplay between the diffusion mechanism and the degrading polymer plays an important role in drug release in a controlled manner [40]. Previous studies have reported that statins using PLGA microspheres as a DDS had a positive effect on osteogenesis around the implant and tooth extraction socket [16, 22]. These studies showed that, in an *in vitro* experiment, FS was gradually released from a PLGA membrane for at least 1 month. In this study, a biodegradable PLGA membrane was used as a carrier to load the drugs (FS) to be released in the healing period, in order to enhance new bone formation in the defect area. However, the optimal dose of statin used for bone formation has not yet been confirmed [16, 20, 22, 55, 56]. No typical finding of inflammation or other adverse symptoms observed in this study indicated that the dose of statin used here (2.4 mg/kg) was in the normal range for bone formation. In previous studies, the concentration of FS ranged from 1.2–3.6 mg/kg, when applied topically to successfully enhance bone metabolism in mice [57, 58]. The systemic bioavailability of FS is low, due to the first-pass effect at the intestinal and/or hepatic level [59]. In this study, FS was loaded locally into a membrane. However, the effect of statin locally applied to a bone defect area was lost within a few days when employed without a suitable DDS [55]. Thus, the selection of an appropriate carrier is essential for the long-term and stable release of statins. Another study has reported that 10 mg/kg of FS with appropriate carrier was effective in enhancing bone formation, even in a single local injection [60]. Our results showed that PLGA–FS compared to PLGA and TM–PLGA–FS compared to TM–PLGA led to higher new bone formation *in vivo*. Therefore, the loading of FS into PLGA can be considered an effective DDS. Further studies are needed to determine the optimal dose of FS and its application to the membrane for GBR.

Spectrophotometric analysis exhibited the stable and long-term continuous release profile of FS released from the PLGA–FS and TM–PLGA–FS membranes over 30 days of *in vitro* experiments. The cumulative release rate tended to increase in both the PLGA–FS and TM–PLGA–FS groups, with approximate release rates of 0.16 and 0.19 μg/50 ml/day, respectively. For polymeric materials, the kinetics of drug release are linked to the physicochemical and morphological properties of the carrier membrane, such as drug diffusion, dissolution, degradation and interactions with the material. In addition, the drug location and its solubility influence the release kinetics and, therefore, the efficiency and efficacy of treatment [61]. In the first 4 days, FS release was observed to be similar between PLGA–FS and TM–PLGA–FS. However, after 5 days of the membranes being immersed in the saline solution, the surface of the PLGA–FS had widened, leading to a higher release of FS than in TM–PLGA–FS. FS was more slowly released from the TM–PLGA–FS, as the TMs maintained the layer of PLGA–FS longer on the surface. After 14 days, the release rate of the TM–PLGA–FS was higher than that of the PLGA–FS. The release kinetics were then shown to be lower over the first 14 days, and continued to increase along with degradation of the membrane. However, the cumulative release rate of FS by PLGA–FS on Day 25 was decreased. The degradation mechanism of the PLGA membranes seemed to influence the controlled release of the FS. Parameters attributed to PLGA degradation and drug release correspond to a variety of physical and chemical systems, such as surface diffusion, bulk diffusion and erosion of the membrane [25, 62]. In addition, the acidic microenvironment may implicate the instability of the release rate of the encapsulated drug from PLGA. The drug-loaded polymer is released mainly through diffusion by the random scission of polymers and by the onset of weight loss. The amount of drug loaded in the matrix and its content effect are attenuated by the rate and duration of drug release [63].

In the present study, bone volumes and bone areas with the PLGA–FS membranes were observed to be larger compared to the PLGA membranes only, as were the TM–PLGA–FS membranes when compared to the TM–PLGA membranes. The application of α-TCP-containing statin has been reported to induce bone regeneration in rat calvaria defects [64]. The amount of variation in the new bone formation and bone remodeling occurred around the defect margin adjacent to the original bone, and growth gradually occurred at the center of the defect site. Minimal bone formation was found in the C group, although the BMD was found to be higher, when compared to the other membrane groups, at 4 and 8 weeks. An abundant amount of bone formation (of about 82%) was observed with 3D analysis in the TM–PLGA–FS membrane at 8 weeks. The defects in this group also exhibited almost complete closure, compared to the TM–PLGA membranes. The results of this study clearly demonstrate the effectiveness of the FS loaded in the TM–PLGA–FS and PLGA–FS membranes for GBR. Other studies have shown that membranes containing a mixture of osteogenic factors can stimulate bone regeneration [37, 65].

Although the bone volume and bone area in the TM–PLGA–FS group were shown to be higher, with significant differences compared to the other groups, the BMD values were found to be lower in this group. It can be speculated that the new bone formation takes time to mineralize, as a lot of nutrients and oxygen may be required. Consequently, the vasculature is developed along the newly formed bone, and the mineral apposition might be incomplete; thus, the BMD is decreased. From a histological analysis, more new bone in contact with original bone in a horizontal growth direction was observed in all the groups. It was supposed that statin may control the expression of factors, such as BMP-2, to differentiate osteoblasts from mesenchymal cells in the periosteum to form new bone. A previous study has reported that calcium phosphate containing FS injected into the periosteum of calvaria regenerated bone formation in a vertical direction [20]. However, despite the function of the FS-loaded degradable PLGA membranes to induce bone regeneration, they also successfully complemented the capability of TMs to maintain the space and prevent the ingrowth of soft tissue through the large pores of TMs. A non-degradable titanium mesh tends to be used in the case of larger bone augmentation, due to its mechanical stability. For large bone augmentation procedures, soft tissue dehiscence and subsequent graft infection are some of the major complications. As described above, it has been reported that statins have positive anti-infection and soft tissue wound-closure effects. These characteristics of statins, therefore, fit the requirements of GBR membranes.

## Conclusions

The results of this study suggested that the new bone formation underneath TMs layered between FS-loaded PLGA was enhanced by the mechanical properties of the TMs and the release of drugs from the FS-loaded PLGA membrane. However, further study is needed to improve the long-term stability, mechanical strength, and concentration of FS-loaded PLGA and TM-PLGA membranes for successful regeneration. A limitation of this study was the difference between this rat model and a real clinical situation. In the considered model, young rats in a growth period were used, whereas GBR treatment is mainly applied to adults. Within the shortcomings of the present study, TMs layered between FS-loaded PLGA membranes show potential for the promotion of bone formation using the GBR technique.

## Author contributions

A.F. conducted in conceptualization (lead), investigation (equal), methodology (equal), validation (equal), writing––review and editing (lead), funding acquisition (lead); Y.D.R. carried out in conceptualization (equal), data curation (lead), formal analysis (lead), investigation (lead), methodology equal), writing—original draft (lead); Y.A. conducted in funding acquisition (supporting), software (supporting), supervision (lead), validation (supporting), writing—review & editing (equal); K.K. carried out in project administration (lead) and supervision (supporting).

## Funding

This study was conducted with financial assistance from the International Team for Implantology, Young Faculty Mentoring Program to K.K. and A.F. and the Japanese Society for Promotion of Science to A.F. (20K10055).

*Conflicts of interest statement*. K.K. belongs to the Division of Advanced Dental Devices and Therapeutics, Faculty of Dental Science, Kyushu University. This division is endowed by GC Corporation, Tokyo, Japan. However, GC Corporation had no specific roles in this study. All other authors state that they have no conflicts of interest.
